# ^1^H-detected solid-state NMR of proteins entrapped in bioinspired silica: a new tool for biomaterials characterization

**DOI:** 10.1038/srep27851

**Published:** 2016-06-09

**Authors:** Enrico Ravera, Linda Cerofolini, Tommaso Martelli, Alexandra Louka, Marco Fragai, Claudio Luchinat

**Affiliations:** 1 Magnetic Resonance Center (CERM), University of Florence, and Interuniversity Consortium for Magnetic Resonance of Metalloproteins (CIRMMP), Via L. Sacconi 6, 50019 Sesto Fiorentino (FI), Italy; 2 Department of Chemistry “Ugo Schiff”, University of Florence, Via della Lastruccia 3, 50019 Sesto Fiorentino (FI), Italy; 3 Giotto Biotech S.R.L., Via Madonna del Piano 6, 50019 Sesto Fiorentino (FI), Italy

## Abstract

Proton-detection in solid-state NMR, enabled by high magnetic fields (>18 T) and fast magic angle spinning (>50 kHz), allows for the acquisition of traditional ^1^H-^15^N experiments on systems that are too big to be observed in solution. Among those, proteins entrapped in a bioinspired silica matrix are an attractive target that is receiving a large share of attention. We demonstrate that ^1^H-detected SSNMR provides a novel approach to the rapid assessment of structural integrity in proteins entrapped in bioinspired silica.

It is the future strategy in biotechnology to use the processes of nature and not only those of chemistry to create biomaterials. The advantage of natural processes is that they occur under physiological conditions, i.e.: in aqueous environment, around neutral pH and at room temperature and ambient pressure. In this light, such processes are promising candidates for a green-based industry. One interesting class of biomaterials is bio-inspired silica, where the polycondensation of silicic acid is directed by the presence of a polycationic templating molecule or by enzymes^1^,^2^,^3^,^4^,^5^,^6^. *In vitro*, large amounts of proteins can be properly encapsulated in silica by simply mixing cationic catalyst with the silicic acid and the protein itself^7^ or, to increase the immobilization yield, the protein can be fused to a biosilica-promoting peptide^8^,^9^,^10^,^11^,^12^,^13^,^14^. The materials obtained by such natural processes can be easily tuned into different morphologies by the use of slight perturbations of the experimental conditions^10^,^15^, and can be easily endowed with peculiar chemical properties, such as fluorescence or catalytic activity by the incorporation of proteins^9^. In turn, proteins that are entrapped in a silicic matrix become more stable and easily reusable^16^,^17^. Despite such a vast applicability, the atomic-level structural characterization of such systems is still far from being routine; indeed, the first solid-state nuclear magnetic resonance (SSNMR) spectra of a biosilica entrapped enzyme were published by us only recently^18^. However, such approach relies on ^13^C detection and thus is intrinsically sensitivity-limited, requiring several milligrams of material for a thorough characterization^19^. We have demonstrated that sensitivity can be dramatically improved by means of Dynamic Nuclear Polarization; and in spite of the sizable reduction in spectral resolution intrinsic of DNP at low temperature, it was possible to observe signals of the properly-folded protein^20^. Of note, in a recent paper, DNP was used to detect the signals from the biosilica-forming peptide PL12, also detecting correlation of silica with lysine sidechains^21^. Another recent paper used DNP to study the proteic component of the biosilica of intact diatoms^22^.

Yet, another option is available for increasing the sensitivity in the detection of enzymes entrapped in bioinspired-silica, and this strategy also has the intrinsic advantage of a more informative chemical shift distribution: the acquisition of ^1^H-detected experiments on ^15^N-labelled samples. Indeed, the development of fast MAS (>50 kHz) and the development of high-field magnets (>18T) allows for proton-detected techniques, because the frequency of the dipolar interaction stays constant but its effect is mitigated by high field and fast MAS is able to accomplish their averaging. This brings into SSNMR^23^,^24^,^25^,^26^,^27^,^28^,^29^,^30^,^31^,^32^,^33^,^34^,^35^,^36^ many of the procedures for assignment and collection of structural restraints routinely applied in solution NMR since several years^37^. Higher fields and faster spinning made it even possible to record ^1^H-^1^H correlation experiments^38^. Recently, ^1^H-detection based experiments were applied to the study of bone^39^. It is important to remark that even though ^1^H-detection has been introduced in solid state NMR already since several years^23^,^24^, it is relevant to understand which samples are amenable for its application. Herein, we demonstrate that proton-detected solid-state NMR, enabled by fast magic angle spinning (60 kHz) and high magnetic field (20 T), is feasible for bioinspired-silica-entrapped proteins. In particular, we show that basic ^1^H-^15^N correlation spectra^29^,^40^ can be acquired in fully protonated, ^15^N-labelled, samples of two proteins, namely green fluorescent protein (GFP) and the catalytic domain of matrix metalloproteinase 12 (catMMP12), both in fusion with the biosilica-promoting R5 peptide (SSKKSGSYSGSKGSKRRIL) at the C-terminus^41^.

## Results and Discussion

**Figure 1:**
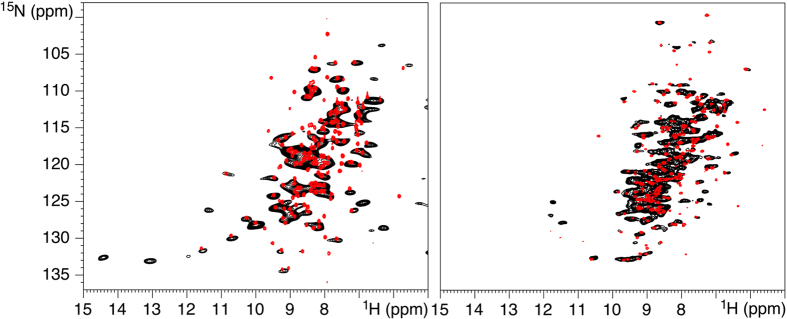
2D ^1^H-^15^N HSQC spectra of catMMP12-R5 (left) and GFP-R5 (right) showing the comparison between the solid-state spectrum (black) and the solution-state spectrum (red). ^1^H-linewidths are about 240 Hz for catMMP12-R5 and 140 Hz for GFP-R5.

The solid state NMR 2D ^1^H-^15^N HSQC spectra of fully protonated GFP-R5 and catMMP12-R5 (Fig. 1) could be acquired in 4 h 34′ and 10 h 36′ respectively on 3 mg of sample, including the bioinspired-silica matrix. The comparison with solution state 2D ^1^H-^15^N HSQC is immediate and reveals that the vast majority of resonances is preserved, confirming the preservation of the structural integrity of the protein (Fig. 1).

**Figure 2:**
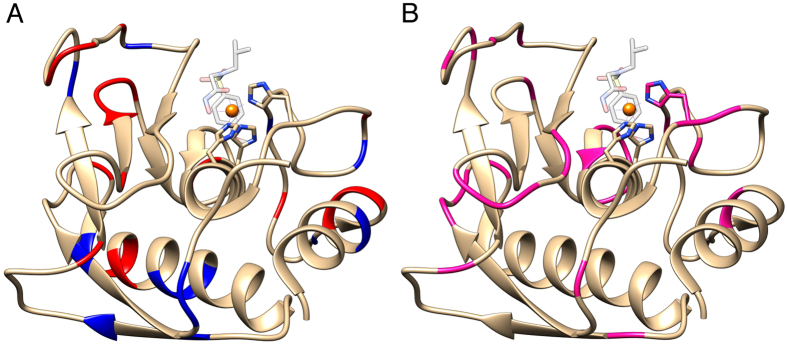
(**A**) The X-ray structure of catMMP12 (1RMZ^42^, which does not carry the R5 fusion) showing the charged residues in blue (positive) and red (negative). (**B**) The structure of catMMP12 with the residues showing no signals shown in magenta. Apparently, they tend to cluster close to loops or in proximity of charged residues, which can interact with the biosilica matrix.

An interesting observation is that a number of resonances could not be observed for MMP12 in the solid state NMR spectrum (Fig. 2 and Table 1). These resonances cluster at, or near to, loops and charged residues. This can be due to two extreme possibilities, i.e.: static disorder or mobility. The first causes heterogeneous broadening of the resonances, the second makes the dipolar-based polarization transfer inefficient. The negligible INEPT-based signal observed previously^18^, suggests that the prevailing effect is static disorder induced by charge interactions with the silica matrix, or by conformational freezing of loops induced by the the presence of the amorphous matrix. This conclusion is further supported by the observation that the residues with missing resonances in the ^1^H-detected experiments are always found (with the only exception of L147) in the ^13^C-based experiment. F237 is not observed in the ^13^C-based strategy, but is observed in the ^1^H-based strategy, whereas its neighboring M236 is not. Finally, residues D171 and T205, which flank missing stretches in the ^1^H-based strategy are even not observed in solution-NMR ^13^C-detected experiments. Taken together, these observations suggest that there is no correlation in the disappearence of peaks between the ^1^H-detection strategy and the ^13^C-detection strategy, and they support the hypothesis that static disorder is sensed to a lesser extent by ^13^C with respect to ^1^H nuclei. Some resonances, which are in substantial overlap with others, are not clearly identifiable as missing, but their absence can in be inferred on a structural basis. Some additional peaks appear in the solid state NMR spectrum of catMMP12-R5, which are not present in the solution NMR spectrum, at proton/nitrogen chemical shifts of 11.4/126.2, 13.1/ 133.1, 14.4/132.6 ppm respectively. These are ^15^N-aliased signals (unaliased 15N chemical shift values are 166.0, 172.8 and172.5, respectively), which belong to the Hδ1 (or Hε2) of the histidine residues coordinating the two zinc ions. These protons are very weak in intensity in the solution NMR because of exchange with water. Two further signals at proton/nitrogen chemical shifts of 6.3/128.6 and 6.9/125.2 ppm, respectively, that are present in the solid state NMR spectrum, can be related instead to the protons of lysines or arginines side-chains, that are lost in the solution NMR spectrum for the same reason.[Table t2]

It is interesting to observe that, in line with a previous observation, the R5 tail does not appear in the spectrum, most probably because it is disordered and thus extremely broad, as observed by the Drobny’s group^45^. As a remark, while it is possible to observe it for the catMMP12-R5, the R5 tail could not be observed in GFP-R5 solution, under the experimental conditions at which assignment of the WT protein is available, i.e.: pH = 8 and T = 310 K, whereas it reappears at lower temperature, indicating exchange with the solvent in line with previous reports on intrinsically disordered proteins^46^,^47^.

The resolution between the two proteins varies quite significantly (240 Hz and 140 Hz ^1^H-linewidth for catMMP12-R5 and GFP-R5 respectively). On the one hand, this may suggest that the residual mobility of the entrapped protein is different in the two cases, yielding a different averaging of the dipolar interaction; on the other hand this could reflect different degrees of static disorder or different anisotropic bulk magnetic susceptibility as caused by different silica particle size^48^. However it is important to observe that resolution is in a practically useful range even if the protein is completely protonated. This is an observation of remarkable importance because, on the one hand, ^1^H-detection based methods have been proven applicable and successful on a rather vast range of substrates^33^, also when fully protonated^40^,^31^,^19^; but, on the other hand, perdeuteration with more or less extensive back exchange is usually necessary to yield decoupling of the ^1^H dipolar network^33^,^49^. Thus, the remarkably sharp lines observed in the present study on fully protonated proteins guarantees, at least for a preliminary step, to use a relatively inexpensive sample, instead of resorting to the more expensive deuterated samples.

Overall, the present approach presents a number of advantages with respect to the approach based on the comparison of ^13^C-^13^C correlation maps. Brilliant discussion of the advantages of moving to smaller volumes^50^ and ^1^H detection^33^ has been provided, and the solid-state NMR rotor technology is progressing fast^38^, so we will only briefly recapitulate the specific advantages in the present case. First of all, the amount of sample needed is far smaller, yielding a reduction in the sample cost. The comparison here reported is based on the catMMP12-R5 sample, which has an encapsulation efficiency of 1.2 nmol of protein per μmol of silica^41^. The total amount of sample for the current work is 3 mg, corresponding to the 1.7 μl of the rotor active volume of a 1.3 mm Bruker rotor; whereas for a 4 mm rotor with CRAMPS inserts, the amount of sample needed to fill a 50 μl volume was over 80 mg^41^. As we are referring to a protein composite, it is rather more instructive to compare the starting protein solutions. The starting protein solution for the sample used in the present work was 400 μl at 45 mg/mL concentration of ^15^N-labeled protein (estimated cost 100 €, not including manpower), whereas the protein solution used for the sample preparation in reference^41^ was 2 mL at 45 mg/mL concentration of ^13^C^15^N protein (estimated cost 900 €, not including manpower), which corresponds to an order of magnitude difference in sample cost.

Another important point is the amount of measurement time that is needed: a ^1^H-start/^1^H detect experiment is expected to be roughly a factor 2.5 more sensitive than a ^1^H-start-^13^C detect experiment, neglecting relaxation effects and assuming 100% efficiency in each transfer step; the amount of experiments to be acquired in the indirect detection is about a factor 6 smaller and the full-rotor sensitivity of a solenoid coil (including the higher efficiency but also the reduction of sample) on passing from a 4 mm to a 1.3 mm rotor is roughly reduced by a factor 9.5 51,50, which would yield about a factor 2 increase in sensitivity, corresponding to about a factor 4 reduction in experiment time, thus in experiment price. These consideration are qualitatively reflected in the present case, as we find that the ^1^H-^15^N HSQC experiment could be acquired in 10 h 36′ for catMMP12-R5 in the current setup, whereas the ^13^C-^13^C DARR experiment reported in reference^41^ was acquired in 45 h 31′. Considering the price of access to high-field NMR solid-state instrumentation, the ^1^H-detection-based approach is about 80% cheaper than the ^13^C-detection based approach.

It is of remarkable importance that the ^13^C-^13^C transfers is likely to be differently efficient between the solution and the solid, resulting in a less straightforward interpretation of the spectral fingerprint of the molecule. Finally, ^1^H is much more sensitive to changes in the local environment.

In conclusion, we here demonstrate that enzymes entrapped in bioinspired silica can be studied by ^1^H-detected solid-state NMR, using a total sample of about 3 mg (including the silica matrix), as compared to previous studies where a larger amount of protein was used; and we were able to show that structural integrity is maintained.

It is expected that a) this approach will make it easier to screen for reaction conditions and/or substrates on a lower amount of sample and in a shorter time, and b) that the use of extensive deuteration will make it possible to perform assignment and structure calculations on a single SSNMR sample, without resorting to solution NMR, although at the price of increased sample production costs.

## Methods

### Solid-state NMR spectroscopy

All spectra were acquired with a Bruker AvanceIII spectrometer operating at 850 MHz ^1^H larmor frequency, equipped with a 1.3 mm probehead tuned to ^1^H-^29^Si-^15^N. Samples were packed into Bruker 1.3 mm zirconia rotors, centerpacked with KFM inserts (3 μl total volume) to avoid dehydration. Sample packing was performed with a ultracentrifugal device (courtesy of Bruker Biospin)^52^. Sample rotation was regulated to 60 kHz via a commercial Bruker MAS II pneumatic unit, and temperature was regulated to 240 K at stator inlet, roughly corresponding to 300 K at the sample. Pulses were 2.5 μs for ^1^H, 3.5 μs ^15^N. Optimal interscan delays are reported along with the spectra. During ^15^N evolution SW_f_TPPM^53^,^54^,^55^,^56^ decoupling was applied at 25 kHz. Water suppression was achieved by a 300 ms MISSISSIPPI pulse train at 25 kHz applied during a z-filter period when the magnetization is stored on ^15^N^29^,^57^. In the solid state HSQC experiment^29^, the polarization transfer steps between different nuclei is accomplished via cross-polarization (CP)^58^. The first CP step, which drives the magnetization from all protons to the nitrogen spins is 1.2-15 ms long; the second step, which must be as short as possible to be specific and not dominated by spin-diffusion, was kept at 300 μs.

### Solution-state NMR spectroscopy

Experiments were performed on samples of the ^15^N isotopically enriched catalytic domain of MMP-12 fused with R5 peptide (catMMP12-R5) at protein concentration of 0.90 mM in water buffered solution (20 mM Tris, pH 7.2, 50 mM NaCl, 0.1 mM ZnCl_2_, 10 mM CaCl_2_, 200 mM AHA). The protein was inhibited with NNGH (N-Isobutyl-N-(4-methoxyphenylsulfonyl)glycyl hydroxamic acid) to reproduce the experimental conditions used in previous studies^18^,^42^,^59^,^60^,^61^. The same experiments were performed on GFP fused with R5 peptide (GFP-R5) at protein concentration of 0.35 mM in water buffered solution (50 mM Tris, pH 8, 300 mM NaCl). The spectrum of catMMP12-R5 was recorded on a Bruker DRX 500 spectrometer equipped with triple-resonance CryoProbe, and temperature was set to 298 K. The spectrum of GFP-R5 was recorded on a Bruker AVANCE 600 spectrometer equipped with a triple resonance room temperature probe, and temperature was set to 310 K. Spectra were processed with the Bruker TOPSPIN software packages and analyzed with the program CARA (Computer Aided Resonance Assignment, ETH Zurich).

### Protein expression and purification

The catalytic domain of MMP-12 (catMMP12) has been expressed as fusion protein with a R5 peptide at the C-terminus as already described^62^.

pET-21a constructs encoding GFP-R5, sequence given below, were transformed into Escherichia coli BL21(DE3) cells which were subsequently cultured in ^15^N-labelled minimal medium (M9). Cells were grown at 310 K, until OD 0.6–0.8 and subsequently induced with IPTG (isopropyl 1-thio-β-D-galactopyranoside) with a final concentration of 0.5 mM for 5 h.

GFP-R5 was then extracted from harvested cells by sonication and subsequent ultra-centrifugation (40 min, 40000 rpm). The protein was first purified from the crude extract with an anionic exchange column (Q-FF 16/10, A buffer: Tris 50 mM pH 8, B buffer: Tris 50 mM pH 8, NaCl 1 M). The GFP-R5 was finally purified with gel filtration using a Superdex 75 26/60 in Tris 50 mM, NaCl 300 mM, pH 8. The concentrations of NaCl was reduced to 50 mM for the preparation of entrapped samples for ssNMR experiments.

The sequence of the GFP-R5 is:

1 MGKVSKGEEL FTGVVPILVE LDGDVNGHKF SVSGEGEGDA TYGKLTLKFI

51 CTTGKLPVPW PTLVTTFGYG LQCFARYPDH MKQHDFFKSA MPEGYVQERT

101 IFFKDDGNYK TRAEVKFEGD TLVNRIELKG IDFKEDGNIL GHKLEYNYNS

151 HNVYIMADKQ KNGIKVNFKI RHNIEDGSVQ LADHYQQNTP IGDGPVLLPD

201 NHYLSTQSAL SKDPNEKRDH MVLLEFVTAA GITLGMDELY K(GSASGGGGS)

251 [SKKSGSYSGS KGSKRRIL]

brackets indicate a linker that connects the protein to the R5 peptide, square brackets denote the R5 sequence (S201-L220 from sil1P from *C. fusiformis*).

## Additional Information

**How to cite this article**: Ravera, E. *et al*.^1^H-detected solid-state NMR of proteins entrapped in bioinspired silica: a new tool for biomaterials characterization. *Sci. Rep.* **6**, 27851; doi: 10.1038/srep27851 (2016).

## Figures and Tables

**Figure 3:**
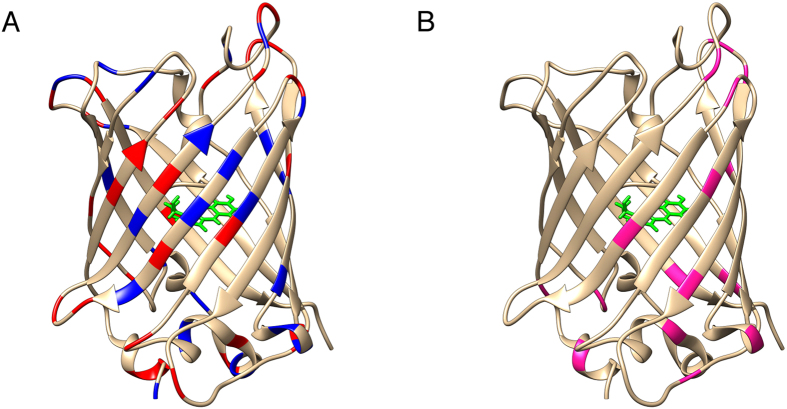
(**A**) The X-ray structure of GFP (2WUR^43^, which does not carry the R5 fusion) showing the charged residues in blue (positive) and red (negative). (**B**) The structure of GFP with the residues showing no signals shown in magenta.

**Table 1:** Residues with missing NH resonances in the catMMP12-R5 ^1^H-^15^N solid state NMR experiment.

**Residue No.**	**Residue Type**	**Flanking sequences**	**Secondary structure Element**
110	R	RKH	Loop
119	N	RINNY	Loop
121	Y	NNYTP	Loop
122	T	NYTPD	Loop
124	D	TPDMN	Loop
147	L	TPLKF	Loop
157	A	GMADI	Loop
166	G	ARGAH	Loop
168	H	GAHGD	Loop
172	H	DFHAF	Loop
176	G	FDGKG	Loop
185	F	HAFGP	Loop
186	G	AFGPG	Loop
188	G	GPGSG	Loop
189	S	PGSGI	Loop
190	G	GSGIG	Loop
201	F	EDEFW	Loop
206	H	TTHSG	Loop
207	S	THSGG	Loop
208	G	HSGGT	Loop
209	G	SGGTN	Loop
211	N	GTNLF	Loop
213	F	NLFLT	Helix
228 (active site ligand)	H	LGHSS	Loop
229	S	GHSSD	Loop
236	M	AVMFP	Loop
241	K	TYKYV	Loop
254	D	ADDIR	Helix

The resonances assignment reported in the bmrb under the accession code 6444^42^ has been used for the interpretation of the spectra. Also for GFP some resonances could not be observed, again maily in proximity of charged residues and loops (Fig. 3 and Table 2).

**Table 2 t2:** Residues with missing NH resonances in the GFP-R5 ^1^H-^15^N solid state NMR experiment.

**Residue No.**	**Residue Type**	**Flanking sequences**	**Secondary structure Element**
7	L	EELFT	Helix
37	A	GDATY	Loop
83	F	HDFFK	Helix
97	T	ERTIS	Strand
104	G	DDGNY	Loop
110	A	TRAEV	Strand
129	D	GIDFK	Loop
130	F	IDFKE	Loop
157	Q	DKQKN	Helix
167	I	FKIRH	Strand
173	D	IEDGS	Loop
175	S	DGSVQ	Loop
185	N	QQNTI	Strand
194	L	GVLLD	Loop
200	Y	NHYLS	Strand

The resonances assignment reported in the bmrb under the accession code 5666^44^ has been used for the interpretation of the spectra.
